# Dietary Interventions Reduce Traditional and Novel Cardiovascular Risk Markers by Altering the Gut Microbiome and Their Metabolites

**DOI:** 10.3389/fcvm.2021.691564

**Published:** 2021-07-14

**Authors:** Amrita Vijay, Stuart Astbury, Louca Panayiotis, Francine Z. Marques, Tim D. Spector, Cristina Menni, Ana M. Valdes

**Affiliations:** ^1^School of Medicine, University of Nottingham, Nottingham, United Kingdom; ^2^Department of Twin Research, King's College London, London, United Kingdom; ^3^Hypertension Laboratory, School of Biological Sciences, Monash University, Melbourne, VIC, Australia; ^4^Heart Failure Research Laboratory, Baker Heart and Diabetes Institute, Melbourne, VIC, Australia

**Keywords:** dietary intervention, short-chain fatty acids, gut microbiome, CVD risk factors, fibre, omega-3 fatty acids

## Abstract

**Aims:** The current study investigates the role of diet in mediating the gut microbiome-cardiovascular association which has not yet been explored in humans.

**Methods and Results:** Using a two-arm dietary intervention study in healthy participants (*N* = 70), we assessed the effects of omega-3 and fibre supplementation on gut microbiome composition and short-chain fatty acid (SCFA) production. We then investigated how changes in gut microbiome composition correlated with changes in traditional cardiovascular risk factors (cholesterol, triglycerides, blood pressure), cytokines, and novel validated markers such as GlycA and ceramides, previously linked to CVD incidence and mortality. Both interventions resulted in significant drops in blood pressure, cholesterol, proinflammatory cytokines, GlycA and ceramides (all *P* < 0.05). Decreases in the atherogenic low-density lipoprotein triglyceride fraction, in total serum cholesterol were correlated with increases in butyric acid-production [β(SE) = −0.58 (0.06), *P* < 0.001; −0.53 (0.04), *P* < 0.001] and nominally associated with increases in some butyrogenic bacteria. Drops in GlycA were linked to increases in *Bifidobacterium* [β(SE) = −0.32 (0.04), *P* = 0.02] and other SCFAs including acetic acid [β(SE) = −0.28 (0.04), *P* = 0.02] and propionic acid [β(SE) = −0.3 (0.04), *P* = 0.02]. Additionally, we report for the first-time reductions in specific ceramide ratios that have been shown to predict CVD mortality and major adverse cardiovascular events such as d18:1/16:0, d18:0/24:0, and d18:1/24:1 which were associated with the reduction in the abundance in *Colinsella* and increases in *Bifidobacteriuim* and *Coprococcus 3* and SCFAs (all *P* < 0.05).

**Conclusion:** Overall, these findings support the potential of using simple dietary interventions to alter validated biomarkers linked to cardiovascular risk via the gut microbiome composition and its metabolic functions.

## Introduction

Cardiovascular diseases (CVDs) remain the main cause of mortality in most Western countries (1, 2). Inflammation and insulin resistance/type 2 diabetes (T2D) are strongly influenced by the gut microbiome composition (3, 4), and have been shown to be predictive factors for CVDs (5–7). Other traditional risk factors for CVD include elevated body mass index, high blood pressure, smoking, increased low-density lipoprotein (LDL) and cholesterol levels, many of which have been similarly associated to the gut microbiome (8). Current guidelines endorse focusing on circulating cholesterol and non-specific inflammatory markers as biomarkers for CVD (9). Recent data show strong links between each of these pathways with circulating biomarkers such as plasma ceramides (complex lipids that play a central role in cell membrane integrity, cellular stress response, inflammatory signalling and apoptosis) and glycosylated acute phase proteins (10–12). Multiple lines of evidence support the association of plasma ceramide levels with adverse cardiovascular outcomes and mortality in patients with prior CVD (10, 13, 14). GlycA, on the other hand (15) and has been shown to be associated with incident CVD, incident and prevalent type 2 diabetes, suggesting that GlycA may also serve as a useful biomarker for assessment of cardiovascular disease risk as well as risk of progression to Type 2 diabetes (T2D) (16–18).

The role of dietary interventions on these two types of markers of CVD risk has not been explored.

A recent systematic review of randomised clinical trials (RCTs) indicated that dietary fibre intake reduces blood pressure, serum cholesterol and insulin resistance (19). Indeed, dietary fibre, through modulation of the gut microbiome, has been shown to reduce blood pressure and cardiac dysfunction in animal models (20, 21). This is due to the production of gut microbiota-derived metabolites such as short-chain fatty acids (SCFAs), which are produced from dietary fibre fermentation by bacteria. SCFAs have been shown to be causal to lower insulin resistance and lower inflammation (22–25), and reduce blood pressure (20, 21, 26, 27). Likewise, omega-3 has been shown, at least in the short-term, to reduce CVD risk factors (28–30) and to influence gut microbiome composition (29, 31, 32). Although previous studies have shown a cardio-protective effect of the Mediterranean diet (33), the detailed mechanisms involved in the interaction between diet and its components, the gut microbiome and CVD and how these can be modified by therapeutic or dietary interventions are currently unknown.

In the current study, we tested and compared two types of dietary supplementation that can alter the gut microbiome as well as CVD risk, namely omega-3 and inulin (a soluble type of prebiotic fibre) (34), to assess the role of changes in gut microbiome composition and SCFA production on traditional (cholesterol, blood pressure, pro-inflammatory markers) and emerging (Glyc A and ceramides) cardiovascular biomarkers.

## Methodology

### Study Design and Intervention

Inclusion and exclusion criteria have been previously reported (34). Briefly, the study participants were unrelated individuals from the TwinsUK registry, a national register of adult twins recruited as volunteers (35). The study was based on an open trial design with randomisation of study participants into either arm, however the researcher and participants were not blinded to the treatment provided. The participation rate was at 97% with 2 dropouts from start to finish of the 6-week dietary intervention period. Participants who were aged >18, with body mass index (BMI) between 20 and 39.9 kg/m^2^ and had a low habitual fibre consumption (<15 g/day) were included. Participants were screened for habitual fibre intakes based on previously completed food frequency records (36). Participants with metabolic or chronic disease and those taking omega 3 or cod liver oil supplements, antibiotics and anti-inflammatory medications were excluded.

Participants were randomised to take either 20 g of inulin fibre (10 g twice daily) or 500 mg of omega-3 supplements (165 mg of EPA, 110 mg DHA, in gelatine capsules) daily for a period of 6-weeks. Neither participants nor researchers were blinded to the interventions and hence allocation order. The participants were booked in for a follow up visit at the end of the 6-week intervention period. Randomisation was performed using an online software (www.sealedenvelope.co.uk). Compliance to supplementation was monitored via food frequency questionnaires and 24-h food recalls which were collected at three time points during the course of the intervention (i.e., baseline, mid-intervention and at follow-up).

Due to the lack of a control arm in the current study, the results being presented do not indicate causality and are only indicative of the effect of treatment (i.e., fibre and omega 3, respectively) on cardiometabolic traits and their associations with gut microbiome composition. The outcomes analyses in this study are the secondary outcomes from the ethics protocol submitted. The trial was approved by the West Midlands Black Country Research Ethics Committee (18/WM/0066) and is registered under the clinicaltrials.gov database (NCT03442348). Participants provided written informed consent.

### Anthropometric Evaluation

Data relevant to this study include BMI, height and weight which were measured at baseline and follow-up visits.

### Assessment of Blood Pressure

Blood pressure was measured by a trained nurse using either the Marshall mb02, the Omron Mx3 or the Omron HEM713C Digital Blood Pressure Monitor performed with the patient in the sitting position for at least 3 min. At each visit, the cuff was placed on the subject's arm so that it was ~2–3 cm above the elbow joint of the inner arm, with the air tube lying over the brachial artery. The subject's arm was placed on the table or supported with the palm facing upwards, so that the tab of the cuff was placed at the same level of the heart. Triplicate measurements were taken with an interval of ~1 min between each reading, with mean of second and third measurements recorded.

### Metabolomic Analysis

#### Serum Short-Chain Fatty Acids

Serum SCFA were measured by the Mass Spectrometry Department, King's College London using *in-situ* pentafluorbenzylation of the free acid species, followed by GC-NCI-MS determination of the resulting derivatives as described previously (34).

#### Ceramides and Glyc A

Ceramide fractions of interest and Glyc A were measured by liquid chromatography-tandem mass spectrometry (LC-MS/MS) using the commercially available Biocrates kit. Concentrations were calculated using appropriate mass spectrometry software (Sciex Analyst®) and data are imported into Biocrates MetIDQ™ software for further analysis.

#### Lipid Metabolites

Circulating levels of cholesterol and triglyceride fractions from fasting serum samples were measured using the high-throughput 1H-NMR metabolomics platform (Nightingale Health Ltd., Helsinki, Finland; nightingalehealth.com/) (37).

#### Inflammatory Markers

Pro and anti-inflammatory serum markers were measured by Affinity Biomarkers, London using the standardised Human Proinflammatory panel 1 assay kit (cat number K151A0H-1), distributed by Meso Scale Discovery.

### Gut Microbiome Sequencing

Faecal samples were collected by the participant at home and brought to the study visit by the participant using previously provided collection kits and frozen immediately at −80°C until further processing. Stool DNA extraction was carried out according to Goodrich et al. (38) using 100 mg of the stool sample. There was no homogenisation prior to this step. Gut microbiome composition was determined by 16 S rRNA gene sequencing carried out as previously described (39). Briefly, the V4 region of the 16S rRNA gene was amplified using universal primers 355F (CCAGACTCCTACGGGAGGCAGC) and 806R (GGACTACHVGGGTWTCTAAT). Amplified DNA was sequenced on the MiSeq platform (Illumina, 300 bp paired-end reads). Read filtering and clustering was carried out using the MYcrobiota pipeline. Briefly, chimeric sequences were filtered using the VSEARCH algorithm within Mothur, and reads were clustered into operational taxonomic units (OTUs) using closed-reference clustering against the SILVA database v132 based on a 97% similarity. Diversity metrics (Shannon index observed OTUs and Unweighted UniFrac) were calculated by rarefying the OTU table down to 7,000 sequences per sample 50 times and taking the average. These analyses were carried out in QIIME2 (v2018.11).

### Statistical Analysis

The study was designed to achieve 32 individuals per arm by follow-up, which was achieved on average. The minimum effect size with 80% power at the alpha = 5% level that can be achieved with this number is beta = 0.45 in units of standard deviation of any of the quantitative measures and this increases to 0.79 units of standard deviation for *p* < 0.001.

All statistical analyses were carried out in R v3.5.2. OTUs with a relative abundance of <0.1% in every sample were removed, and zero inflated relative OTU abundances were inverse normal transformed before further analyses. We investigated the different effects of fibre and omega-3 interventions on changes in OTU abundances (at the genus level) by running general linear models, with change in OTU abundance as the outcome and fibre/omega-3 intervention as the exposure. We not only compared differences between baseline and follow-up but also tested for the correlations in the longitudinal changes between various traits (e.g., SCFAs and cardiovascular markers), therefore we adjusted the analyses for age, gender and BMI (as potential confounders) and also for multiple testing using false discovery rate (FDR q < 0.05). We further investigated the associations between changes in OTUs and SCFAs between baseline and follow-up with changes in CVD traits. General linear models were also carried out to assess for association between the cardiovascular traits using gut microbiota composition and SCFAs as independent predictors along with covariates such as age, sex, BMI and multiple testing (FDR q < 0.05). To formally assess the contribution of short chain fatty acids to the association between significant OTUs and CVD risk factors, we conducted mediation analysis using the “mediation” package in R61 where we considered OTUs as mediator for the causal effect of the gut microbiota on CVD risk factors. Mediation analysis was performed using short chain fatty acids that were associated with both OTUs and CVD risk factors. We used linear mixed effects models (lme4 package in R) for both the mediator and outcome models. For all models, we report the percentage causal mediation effect (ACME), the percentage direct effect (ADE). ACME represents the average size of the effect of the OTUs on CVD risk factors that is mediated by short chain fatty acids, while ADE represents the direct effect of OTUs on CVD risk factors.

## Results

**Figure 1:**
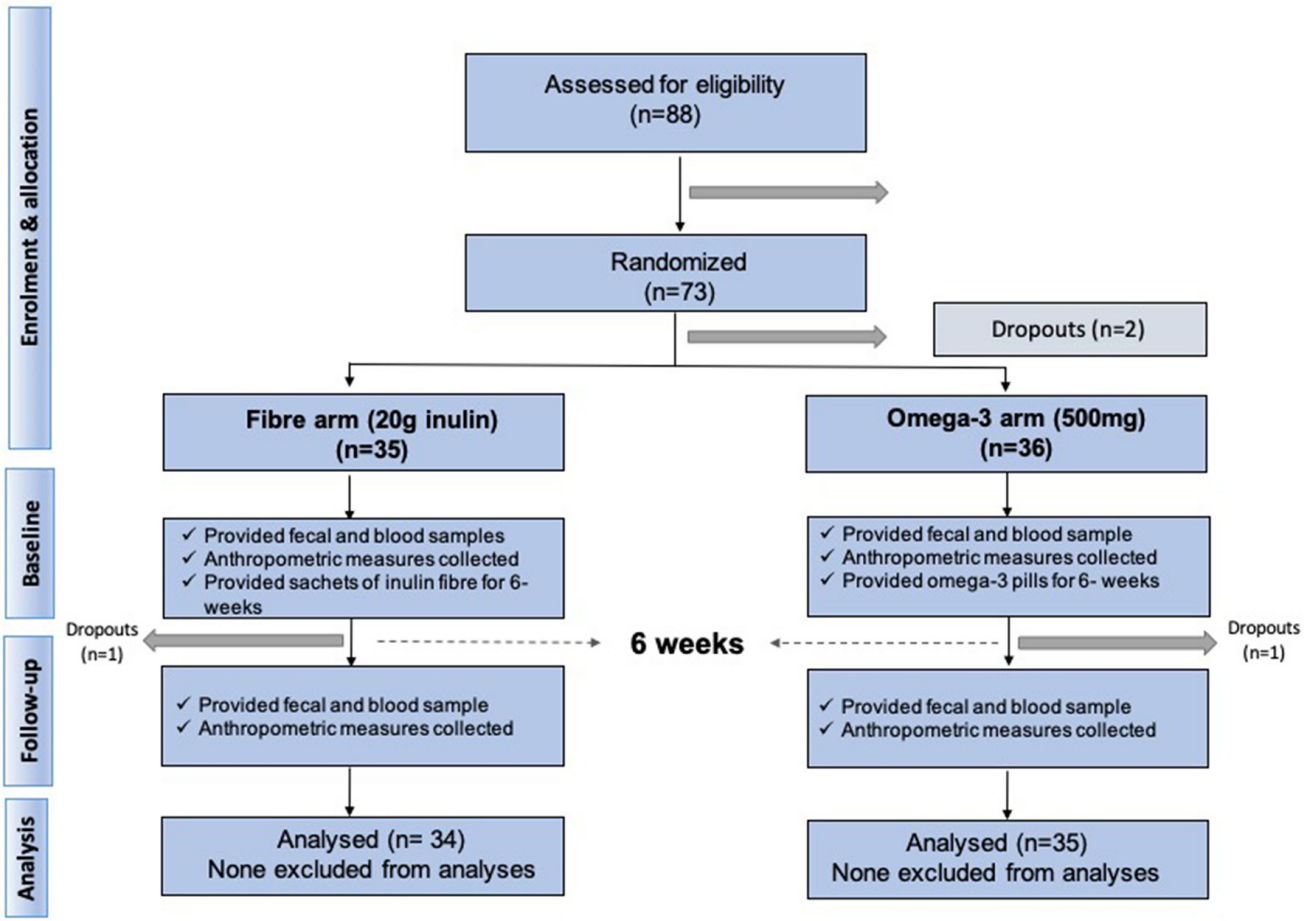
Consort diagram summarising study recruitment and design.

**Figure 2:**
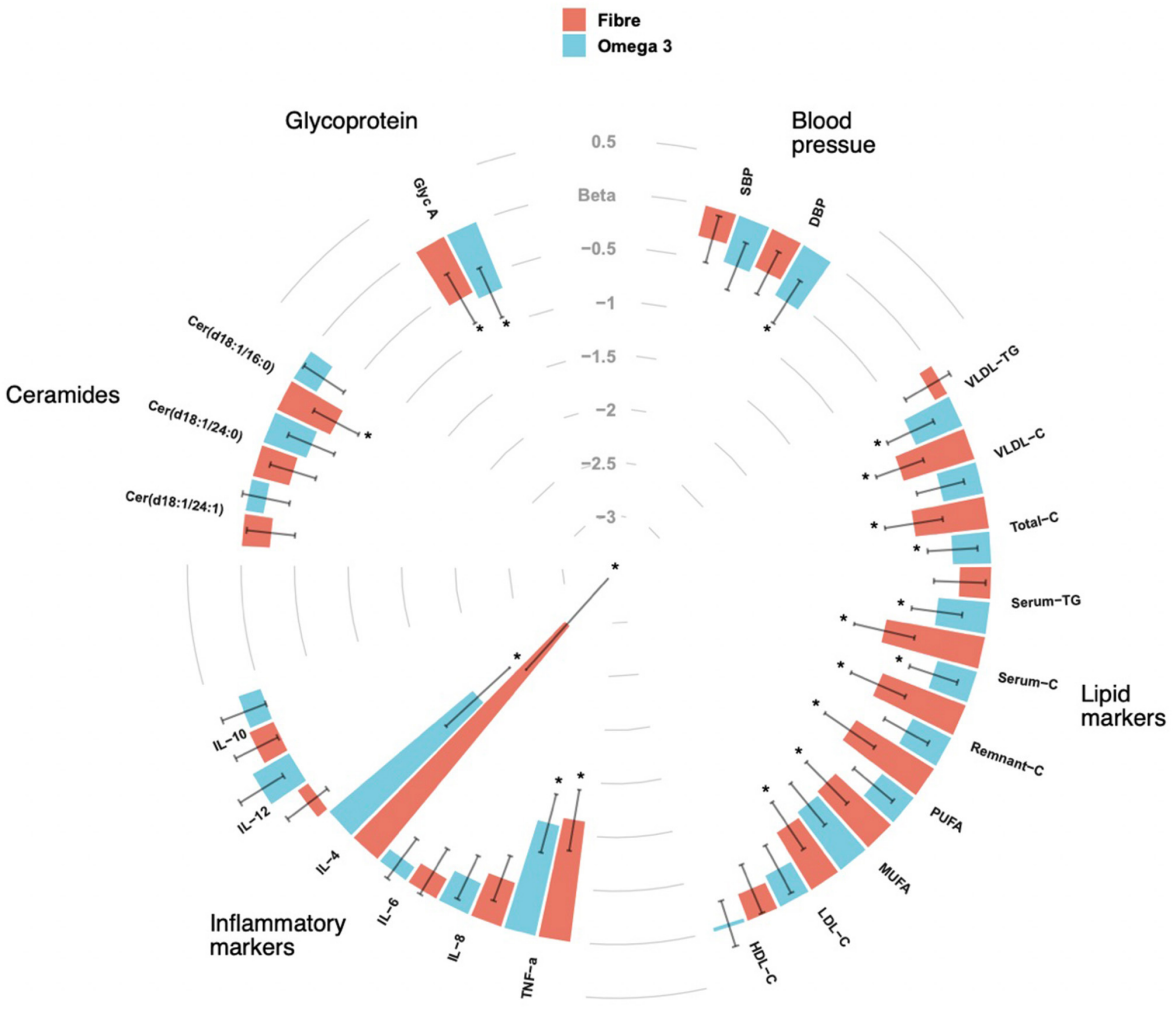
CIRCOS plot showing the association of Fibre and Omega 3 intakes with cardiovascular risk factors such as blood pressure, lipid biomarkers, inflammatory markers, ceramide ratios and Glycoprotein A (Glyc A). Values are beta coefficients from linear models adjusted for BMI, age and gender. *P*-values are adjusted for FDR (* < 0.05).

We included 70 middle aged participants with a mean age 65.2 ± 9.3 (mean ± SD), mainly women (85.7%), slightly overweight (BMI = 26.8 ± 4.3) in a randomised trial of two arms (omega-3 and inulin) as outlined in Figure 1. The baseline characteristics of the study participants and levels of traditional and novel CVD risk factors at the beginning and at end of each intervention are summarised in Table 1. We first investigated whether 6-weeks dietary supplementations had an effect on CVD risk factors. We found significant changes in the levels of traditional risk factors with beta ranging from −2.81 to 0.11 and novel risk factors with beta ranging from −0.74 to −0.26 in the two arms relative to baseline as summarised in Figure 2.

**Table 1:** Characteristics of the study population at baseline and at the end of the 6-week dietary intervention.

	**Fibre**		**Omega 3**	
**Variable**	**Baseline Mean (SD)**	**Follow-upMean (SD)**	**Baseline Mean (SD)**	**Follow-upMean (SD)**
Men/women	5/35 (14.3/85.7%)		3/34 (8.8/91.1%)	
Age (y)	66.83 (9.3)		63.67 (10.76)	
BMI (Wt/Ht^2^)	26.68 (4.4)	26.64 (4.31)	27 (3.740)	26.40 (4.94)
SBP (mm Hg)	134.75 (20.17)	128.41 (17.87)^**^	131.7 (19.42)	124.8 (14.75)^**^
DBP (mm Hg)	76.18 (10.30)	73.07 (8.56)^*^	78.87 (11.96)	72.41 (9.68)^***^
Acetic acid (μmol/l)	43.43 (68.69)	49.10 (10.4)	174.2 (127.8)	180.3 (171.4)
Propionic acid (μmol/l)	10.13 (0.79)	11.97 (5.62)	9.71 (5.96)	10.48 (7.52)
Butyric acid (μmol/l)	8.39 (0.73)	11.61 (4.85)^***^	7.06 (4.54)	7.78 (3.52)
Valeric acid (μmol/l)	1.25 (0.52)	1.31 (0.61)	0.99 (0.60)	1.183 (0.58)
Iso-butyric acid (μmol/l)	10.69 (5.70)	14.3 (6.82)^*^	8.47 (5.75)	10.89 (5.42)^**^
Iso-valeric acid (μmol/l)	7.26 (2.45)	9.73 (4.65)^*^	6.94 (3.80)	9.83 (3.20)^**^
IL4 (ng/l)	0.15 (0.09)	0.10 (0.48)^***^	0.10 (0.03)	0.06 (0.03)^***^
IL6 (ng/l)	1.42 (1.22)	1.12 (0.45)	1.96 (5.26)	1.93 (4.46)
IL8 (ng/l)	31.79 (17.19)	23.30 (11.54)^**^	37.07 (28.14)	25.5 (16.47)^*^
IL10 (ng/l)	0.67 (0.53)	0.70 (0.66)	0.50 (0.26)	0.61 (0.17)
TNFα (ng/l)	3.37 (0.89)	2.58 (0.79)^***^	3.36 (2.00)	2.59 (1.06)^*^
Serum cholesterol (mmol/l)	5.136 (1.03)	4.347 (1.06)^**^	5.207)0.88)	4.717 (0.55)^*^
Serum Triglycerides (TG) (mmol/l)	1.01 (0.37)	1.02 (0.39)	1.10 (0.38)	0.92 (0.35)^*^
LDL-C (mmol/l)	0.23 (0.13)	0.17 (0.10)^**^	0.25 (0.13)	0.23 (0.12)
VLDL-C (mmol/l)	0.64 (0.26)	0.52 (0.21)^**^	0.58 (0.24)	0.50 (0.20)^*^
VLDL-TG (mmol/l)	0.558 (0.29)	0.541 (0.33)	0.617 (0.30)	0.488 (0.31)^**^
Glycoprotein A (mmol/l)	1.604 (0.16)	1.517 (0.17)^**^	1.591 (0.18)	1.512 (0.19)^*^
Cer_d18:1/16:0	0.442 (0.06)	0.337 (0.21)^*^	0.452 (0.08)	0.296 (0.21)
Cer_d18:1/24:0	2.636 (0.71)	2.056 (1.32)	2.703 (0.66)	1.917 (1.49)^*^
Cer_d18:1/24:1	0.154 (0.03)	0.111 (0.07)	0.143 (0.02)	0.099 (0.07)

*BMI, Body mass index; SBP, systolic blood pressure; DBP, diastolic blood pressure*.

* *p < 0.05;*

** *p < 0.01;*

*** *p < 0.001*.

*P-values obtained from paired matched t-test between baseline and follow-up unadjusted for covariates and FDR correction*.

*No significant associations (p < 0.05) were seen in any of the baseline measures between the two arms*.

### Traditional CVD Risk Factors

#### Blood Pressure

We observed significant decreases in both systolic and diastolic blood pressure following 6-weeks supplementation with both omega-3 and inulin as shown in Table 1. However, after adjusting for age, sex and BMI, only diastolic blood pressure after Omega 3 intervention was significant [β(SE) = −0.5 (0.23), *P* = 0.02].

#### Serum Lipids

Of the 158 circulating serum cholesterol lipids measured by Nightingale, 58 were significantly different after the fibre intervention, while 51 were significant after supplementation with omega-3 intervention, of which 3 were overlapping adjusting for covariates (age, sex, and BMI) and FDR. The strongest association for both fibre and omega 3 were observed for serum cholesterol [β(SE) = −0.6 (0.26), *P* = 0.001] and remnant cholesterol [β(SE) = −0.6 (0.25), *P* = 0.005]. There were significant decreases seen for serum TG [β(SE) = −0.4 (0.23), *P* = 0.01] and low-density lipoprotein triglyceride (VLDL-TG) [β(SE) = −0.46 (0.23), *P* = 0.03] observed in the omega-3 arm whereas significant drops in LDL-C [β(SE) = −0.6 (0.23), *P* = 0.01] and VLDL-C [β(SE) = −0.6 (0.02), *P* = 0.01] were found in the fibre arm.

#### Inflammatory Markers

We observed decreases in the levels of 2 of the 5 pro-inflammatory cytokines measured namely IL-4 [β(SE) = −2.17 (0.51), *P* < 0.001] and TNFα [β(SE) = −1.08 (0.28), *P* < 0.001] in the fibre and omega-3 arm. We also found significant decreases in the levels of IL-8 in both arms, however, this was not statistically significant after adjusting for age, sex, and BMI.

### Novel CVD Risk Factors

#### Ceramides and Glyc A

In addition to traditional CVD risk factors, we also measured changes in novel fractions of ceramides and glycoprotein A (GlycA) which have both been known to be associated with adverse cardiovascular outcomes. We measured specific ceramide fractions that have shown to predict CVD mortality and MACE and found significant reductions in ceramide serum levels after both interventions. Importantly, different effects were seen with fibre and omega-3 as illustrated in Figure 2. In addition, we also found significant decreases in levels of GlycA in both intervention arms with both fibre [β(SE) = −0.58 (0.26), *P* = 0.02] and omega-3 [β(SE) = −0.66 (0.25), *P* = 0.007] even after adjusting for covariates.

### Associations of Traditional Risk Factors With Gut Microbiome Composition and SCFAs

There were no significant differences in alpha and beta diversities for omega-3 and fibre intervention arms as previously reported (34). However, there were specific OTU's which were significantly associated with omega-3 and fibre interventions, respectively (Supplementary Figure 1).

**Figure 3:**
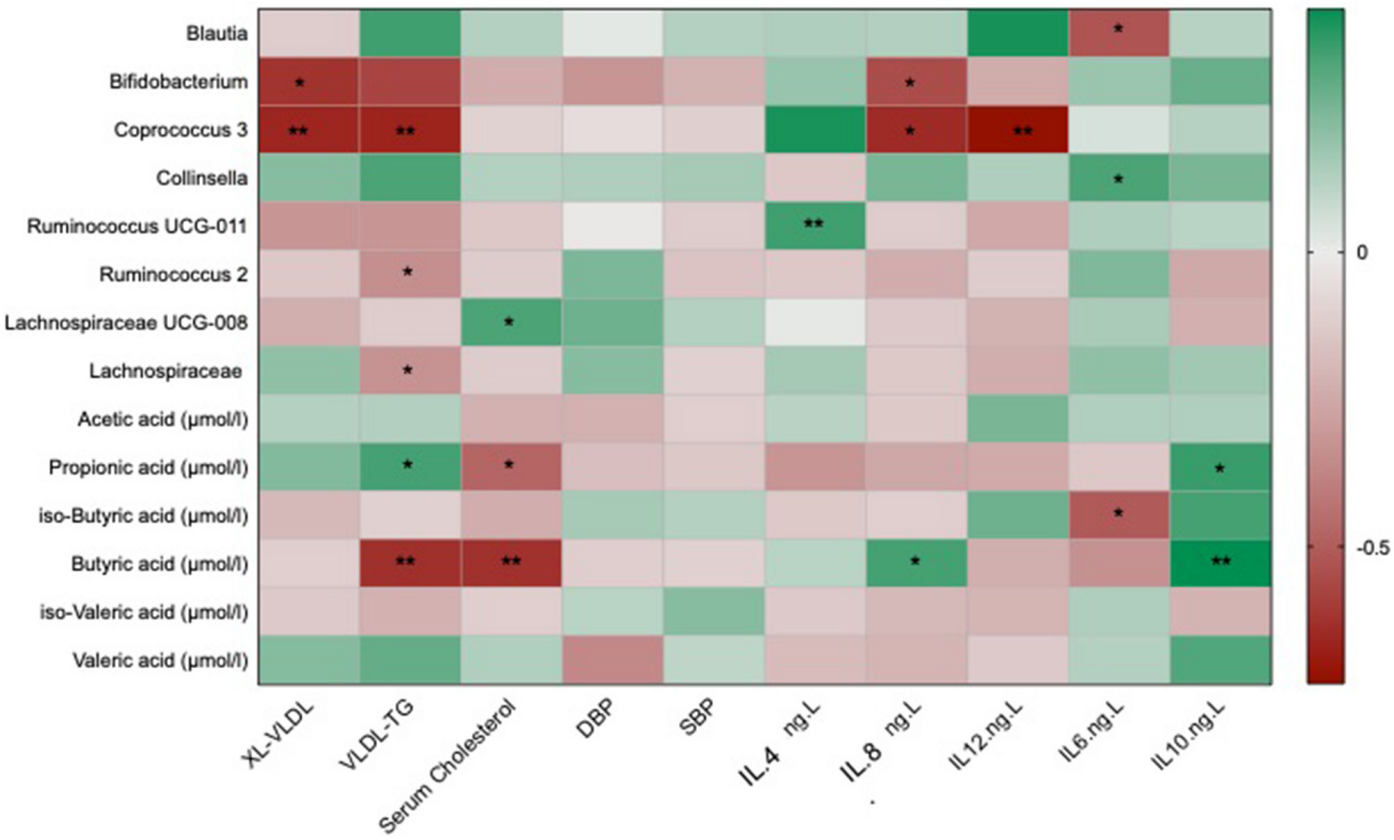
Heat map showing OTUs clustered at genus level and short chain fatty acids associated with the traditional markers of cardiovascular disease which include lipid and cholesterol fractions, blood pressure and inflammatory cytokines. Values are beta coefficients from linear models adjusted for BMI, age and gender. The heat map is colour coded by correlation according to the table legend (green for positive and red for negative correlations). *P*-values are adjusted for FDR (* < 0.05; ** < 0.001).

In order to assess if any of the significant traditional cardiovascular markers were associated with the differential changes in the gut microbiome and its metabolites, we correlated the above markers with all the genera that were significantly altered in one of the two interventions and with changes in short chain fatty acids that have been reported previously (34). After adjusting for covariates and FDR, we found significant negative associations of atherogenic lipid markers such as serum cholesterol and VLDL-TG with *Coprococcus 3* [β(SE) = −0.58 (0.04), *P* < 0.001] and *Bifidobacterium* [β(SE) = −0.37 (0.04), *P* = 0.03] which were both significantly increased in the omega-3 and fibre groups, respectively, as shown in Figure 3. VLDL-TG and serum cholesterol were found to be significantly negatively correlated with butyric acid [β(SE) = −0.58 (0.06), *P* < 0.001; β(SE) = −0.53 (0.04), *P* < 0.001]. The anti-inflammatory cytokineIL-10 was found to be positively associated with SCFAs such as butyric acid [β(SE) = 0.21 (0.03), *P* < 0.001] and propionic acid [β(SE) = 0.18 (0.03), *P* = 0.02]. Blood pressure changes, however, were not found to be significantly associated with microbial species or SCFAs.

### Associations of the Novel Risk Factors With Gut Microbiome Composition and SCFAs

**Figure 4:**
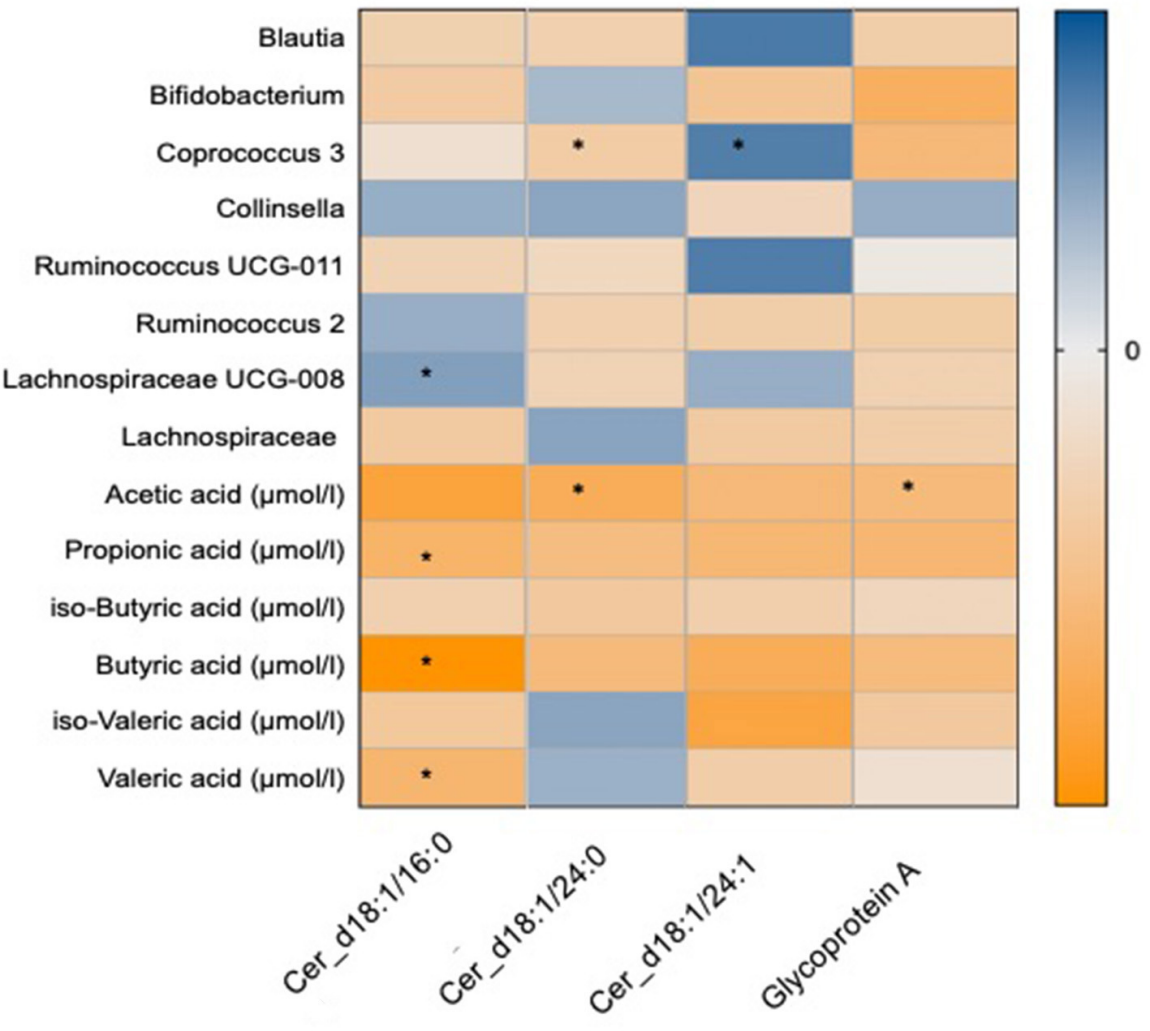
Heat map showing OTUs clustered at genus level and short chain fatty acids associated with the novel markers of cardiovascular disease which include ceramide fractions and glycoprotein A. Values are beta coefficients from linear models adjusted for BMI, age and gender. The heat map is colour coded by correlation according to the table legend (blue for positive and yellow for negative correlations). *P*-values are adjusted for FDR (* < 0.05).

The changes with dietary intervention of a range of ceramide fractions were associated with changes in both microbial genera and SCFAs as shown in Figure 4. Two of the significant OTUs from the omega-3 and fibre groups (34), *Coprococcus 3* was found to be negatively associated with d18:1/24:0 [β(SE) = −0.32 (0.03)]. Furthermore, both d18:1/16:0 and d18:1/24:0 were negatively associated with a range of SCFAs as shown in Figure 4. GlycA was found to be negatively associated with *Bifidobacterium* [β(SE) = −0.32 (0.04), *P* = 0.02] and SCFAs such as acetic [β(SE) = −0.28 (0.04), *P* = 0.02] and propionic [β(SE) = −0.3 (0.04), *P* = 0.02] acids as shown in Figure 4.

### Proportional Variance Explained by the Gut Microbiota and Short Chain Fatty Acids on the Associations With CVD Markers

**Figure 5:**
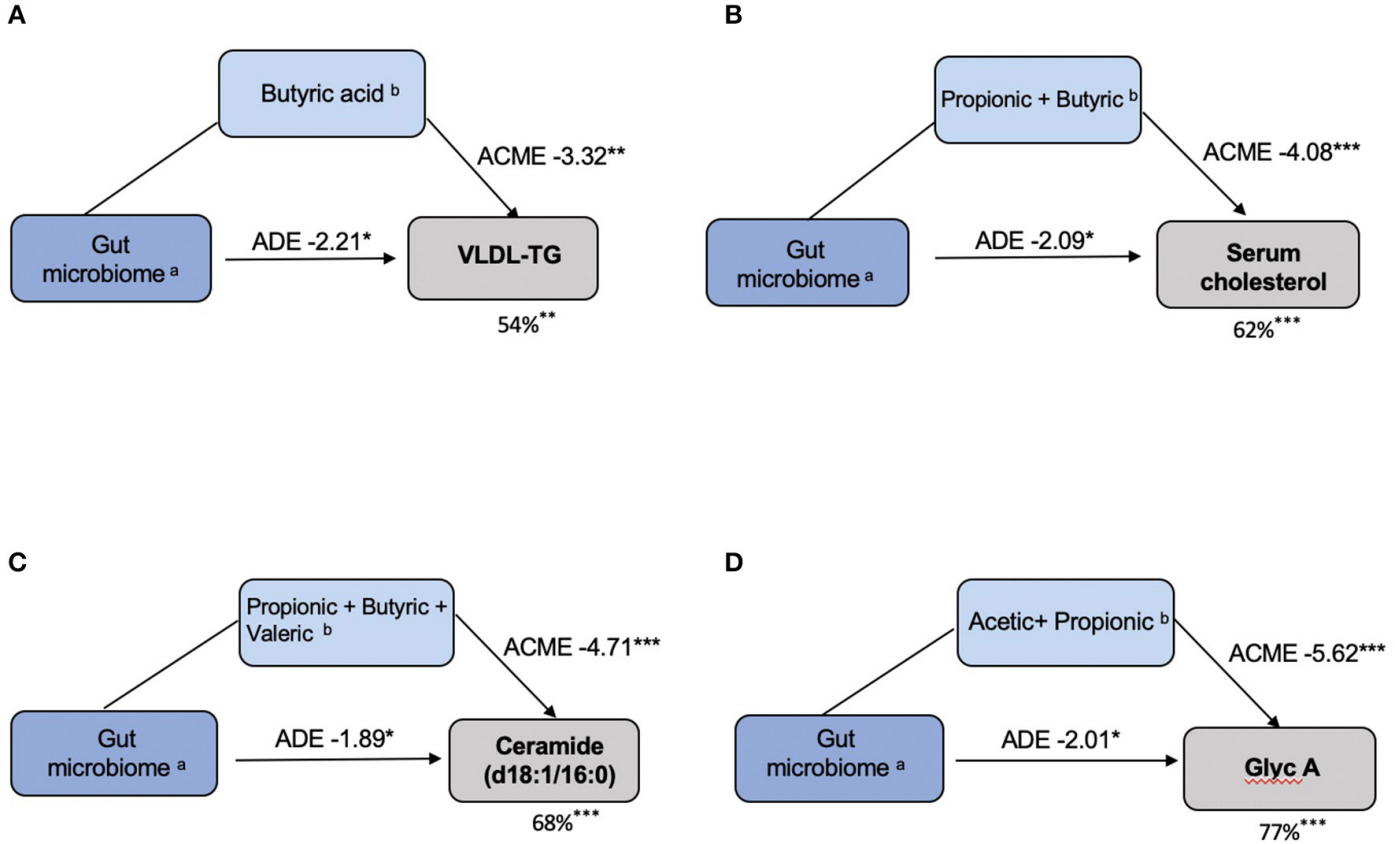
SCFAs mediate the effects of the gut microbiota on CVD markers. **(A)** VLDL–TG: ^(a)^Coprococcus 3; Ruminococcus 2; Lachnospiraceae; ^(b)^Butyric acid. **(B)** Serum Cholesterol: ^(a)^ Lachnospiraceae _UCG-008; ^(b)^ Propionic acid, Butyric acid. **(C)** Ceramide (d18:1/16:0): ^(a)^ Lachnospiraceae _UCG-008; ^(b)^ Propionic acid, Butyric acid, Valeric acid. **(D)** Glycoprotein A: ^(a)^ Bifidobacterium; ^(b)^ Acetic acid, Propionic acid. In each model, the left box represents the causal variable (gut microbiome), the top box is the mediator (SCFA) and the right box is the response (CVD marker). The number by the top arrow represents the average causal mediation effect (ACME) and the one on the bottom arrow is the average direct effect (ADE). The number under the CVD marker box indicates the percentage of mediation. *P < 0.05, **P < 0.01, ***P < 0.001.

We then explored the proportional effect of the gut microbiota and SCFAs on CVD markers that were significantly associated (FDR q < 0.05) with both these parameters. We explored these effects by formal mediation where SCFAs were fitted as mediator of the effect of gut microbiome composition on CVD markers. Overall, we found that SCFAs partially mediated the association between the gut microbiome and CVD markers. SCFAs mediated 54% (*P* < 0.01), 62% (*P* < 0.001), 68% (*P* < 0.001) and 77% (*P* < 0.001) of the effect of VLDL-TG, serum cholesterol, ceramide (d18:1/16:0) and Glyc A, respectively, as shown in Figure 5.

## Discussion

In the current study, we show that supplementation with omega-3 and inulin fibre results in significant reductions in traditional and novel CVD risk factors which are modulated via gut microbiome composition and metabolic function. We show for the first time that increased production of SCFAs due to either fibre or omega-3 dietary interventions results in lower inflammation and lower CVD risk markers. This correlates with changes in bacterial composition, in particular increases in SCFA-producing bacteria such as *Bifidobacterium* and *Coprococcus 3* in the fibre and omega-3 arms as reported previously (34). Our findings are consistent with the recent multicentre Mediterranean diet intervention (33) as well as previous studies that have identified gut microbial signatures and functional metabolites that are strongly associated with cardiometabolic health mediators and dietary habits (40, 41). Moreover, we find that most of the bacterial genera that changed in response to dietary supplementation (34) and whose change (either increased or decreased) relates to improved CVD markers, such as *Ruminococcus, Lachnospiraceae, Blautia*, and *Bifidobacterium*, were also identified in the observational study of CAD (42). The consistency between these studies points out to the robustness of the results and suggests that these findings may have strong translational potential.

Both Omega-3 and fibre have shown similar effects on reducing inflammation and cardiometabolic risk factors, specifically in reducing serum cholesterol levels, pro-inflammatory markers such as IL-4, IL-8 and ceramide ratios such as d18:1/16:0 which has shown to predict CVD mortality. These changes in the fibre arm were, in turn, correlated with increases in butyric and iso-butyric acids which were both increased with fibre supplementation (34).

In addition to the traditional CVD markers, there have been several studies linking ceramides with risk of major cardiovascular events. A previous observational study in humans found that specific gut bacteria might affect atherosclerosis by modulating metabolic pathways of the host including ceramides (42). Our interventional study confirms that changes in gut microbiome composition induced by diet alter proven biomarker ceramide fractions such as d18:1/16:0 and d18:1/24.0 (14, 43). Although not significant, both d18:1/16:0 and d18:1/24:0 were positively associated with *Collinsella*, a genus previously known to be associated with biopsy-proven non-alcoholic steatohepatitis (39). This genus was found to be decreased with increased fibre intake as reported previously (34, 39). Importantly different effects were seen with fibre and omega-3 suggesting different mechanisms which were modulated either by changes in SCFAs or specific OTUs. Several reports have found that ceramide concentrations are significantly decreased by caloric restriction, gastric bypass, aerobic exercise and statin therapy (44–46), however, this is the first study that reports the effects of diet on modifying ceramide ratios that have been previously associated with increased CVD risk.

Our results also showed significant reductions in GlycA levels in both the omega-3 and fibre interventions and that the amount of the drop correlated with increases in acetic and propionic acids and in levels of *Bifidobacterium*. We find in fact that direct the effect of gut microbes on GlycA levels is relatively modest whereas most of the effect is mediated by SCFA levels, consistent with SCFAs being anti-inflammatory. Given the important role of GlycA (i.e., glycosylated acute-phase proteins) in defining cardiovascular and diabetes risk (15), these data suggest that GlycA can be effectively reduced through dietary interventions aimed at increasing circulating levels of SCFAs.

The potent anti-inflammatory properties of SCFAs on immune cell function has been postulated as one of the mechanisms for their cardioprotective effect (47). The cardioprotective effects of SCFAs have been elucidated in previous animal models wherein supplementation with propionate (27) or acetate (21) have resulted in the prevention of organ damage, development of hypertension and heart failure. Furthermore, several studies have demonstrated the anti-inflammatory properties of butyrate and its effects on maintaining host homeostasis. Although their mode of action has yet to be elucidated, SCFAs may also exert their effects via inhibition of histone deacetylases (HDACs) or via specific G-protein–coupled receptors (Gpr) (48, 49). Although there is a substantial body of literature linking SCFAs to CVD risk factors, there is a paucity of studies showing clear association with circulating SCFAs and CVD risk or mortality in humans. Our current study expands on the cardiovascular protective effects of SCFAs in humans, pointing to the important contribution of diet in modulating the anti-inflammatory properties and immune homeostasis of SCFA-mediated effects.

Our study has several strengths, being a randomised intervention it does not suffer from the confounding seen in observational studies. The deep molecular phenotyping assessing well-characterised yet novel cardiovascular markers (ceramides, GlycA) along with inflammatory cytokines and SCFAs allows us to dissect the likely mechanistic links between gut microbiome changes induced by diet and cardiovascular risk markers. Unlike the effects seen through a complex healthy diet intervention, such as the Mediterranean diet, we showed that it is possible to see significant effects both on microbiome composition and cardiometabolic health mediators via simple dietary components. Furthermore, in spite of the lack of a placebo arm this is the first study to show associations between dietary components, gut microbiome and cardiovascular markers. We also note several limitations: the study sample is not large and did not allow us to investigate small effects, only the major microbiome changes. This has nonetheless enabled us to see considerable consistency with previous observational and interventional studies (33, 42). In addition, we did not include a placebo arm in the study which may limit us from identifying causal links between microbiome composition, function and CVD markers. For example, the observed drops in blood pressure parameters in our study may be indicative of random fluctuation over time, a possibility that we are unable to exclude because due to the absence of a placebo arm. We can therefore not assume any causal links with the dietary intervention, although drops in blood pressure (specifically DBP) has also been previously reported amongst normotensive adults show significant drops in DBS following a diet enriched with omega 3 (50). Furthermore, a lack of the placebo arm with no effects on the gut microbiome and participants were not blinded. This is, however, a common limitation of dietary interventions. Inclusion of non-digestible non-fermentable carbohydrate placebo such as the one used by Deehan et al. (51) might have removed any confounding introduced by these two aspects.

The current study highlights the potential of simple dietary supplementations in order to modulate cardiometabolic markers which can be explained in part by changes in the gut microbiome composition and mediated by increases in SCFA production. Future directions involve understanding the specific role of the main SCFAs in the cardiovascular risk pathways and designing carbon sources (forms of fibre) that can increase selectively different SCFAs (51).

## Data Availability Statement

The raw data supporting the conclusions of this article will be made available by the authors, without undue reservation.

## Ethics Statement

The studies involving human participants were reviewed and approved by the West Midlands Black Country Research Ethics Committee (18/WM/0066). The participants provided their written informed consent to participate in this study.

## Author Contributions

AMV and TS: conceived and designed the experiments. AV and SA: analysed the data. LP and CM: contributed materials/analysis tools. AV and AMV: wrote the manuscript. AMV, CM, FM, SA, and TS: revised the manuscript. All authors contributed to the article and approved the submitted version.

## Conflict of Interest

TS is co-founder of Zoe Global Ltd. AMV is a consultant for Zoe Global Ltd. The remaining authors declare that the research was conducted in the absence of any commercial or financial relationships that could be construed as a potential conflict of interest.
