# Supplementary material for: Dietary Interventions Reduce Traditional and Novel Cardiovascular Risk Markers by Altering the Gut Microbiome and Their Metabolites
Source: Front Cardiovasc Med. 2021 Jul 14;8:691564. doi: 10.3389/fcvm.2021.691564 (PMC8319029; doi:10.3389/fcvm.2021.691564)
Supplement: Supplementary file 1 [file Data_Sheet_1.PDF]

Supplementary files

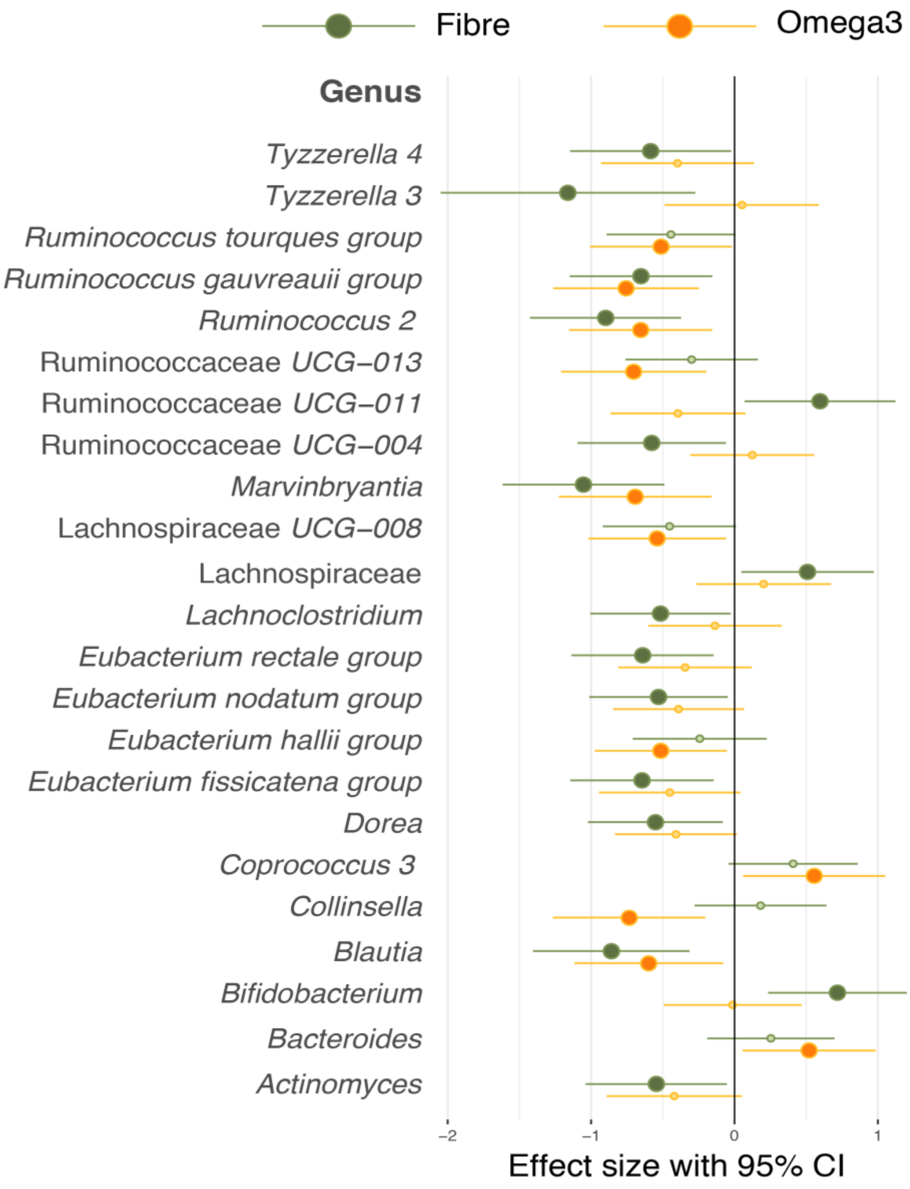

Figure 1: Forest plot of effect sizes with 95% confidence intervals showing association of all significant (FDR adjusted p value <0.05) OTUs in the Fibre and omega-3 intervention arms. Smaller dots on the fibre and omega-3 arm indicate a loss of statistical significance. Association was tested by paired t-tests between baseline and follow-up.
